# Role of placental barrier on trace element transfer in maternal fetal system and hypertensive disorders complicating pregnancy and gestational diabetes mellitus

**DOI:** 10.1186/s12884-023-06183-3

**Published:** 2023-12-16

**Authors:** Ailing Ding, Huimin Wan, Juan Peng, Huizi Wang, Shaodan Zhu, Xudong Dong

**Affiliations:** 1 Faculty of Life Science and Technology, Kunming University of Science & Technology, Kunming, 650500 China; 2 Medical school, Kunming University of Science and Technology, Kunming, 650500 China; 3 The Obstetrical Department of the First People’s Hospital of Yunnan Province, The Affiliated Hospital of Kunming University of Science and Technology, Kunming, 650032 China

**Keywords:** Trace elements, Placental barrier, Hypertensive disorders complicating pregnancy, Gestational Diabetes

## Abstract

**Background:**

Hypertensive disorders complicating pregnancy (HDCP) and gestational diabetes mellitus (GDM) can affect the placental barrier function to varying degrees. However, current studies show that the transfer and distribution characteristics of trace elements in the maternal-fetal system are still unclear. This study investigated the effect of the placental barrier on the transfer of trace elements from mother to fetus and its relationship with HDCP and GDM.

**Methods:**

A case-control method was used in this study. 140 pairs of samples were collected; 60 were from healthy pregnant women, and 80 were from patients with pregnancy complications. The contents of trace elements in paired samples were determined by inductively coupled plasma–mass spectrometry (ICP-MS). SPSS software was used to analyze the differences in trace element levels in matched samples of each group. The correlations were analyzed based on Pearson’s correlation factor (r).

**Results:**

The distribution characteristics of Fe content in the pathological group (HDCP group and GDM group) were the same as those in the normal group (umbilical cord blood > maternal blood > placenta), but there was no significant difference in the iron content in maternal blood and cord blood of pathological group. The distribution characteristics of Mn content in the pathological group (placenta > umbilical cord blood > maternal blood) were changed compared with those in the normal group (placenta > maternal blood > umbilical cord blood). In addition, the placental Cr content and cord blood Cr and Ni content of the pathological group were higher than those of the normal group. HDCP placental Cr and GDM placental Fe levels were significantly correlated with the Apgar score.

**Conclusions:**

The transfer of Fe and Mn and the placental barrier function of Cr and Ni in the maternal-fetal system of HDCP and GDM are significantly altered, which directly or indirectly increases the maternal and fetal health risk.

## Background

Trace elements refer to trace elements or nutrients needed by the human body [1]. Human exposure to trace elements comes from a variety of sources, including air, drinking water and food [2]. Trace elements zinc (Zn), copper (Cu), iron (Fe), manganese (Mn), nickel (Ni) and chromium (Cr) are essential to maintain normal cell function and physiology [3]. Abnormal levels of these elements have been linked to various diseases, especially in pregnant women and fetuses.

Pregnancy is a very complex process; trace elements will have different degrees of adverse effects on the fetus, and the participation and coordination of trace elements are inseparable. Trace elements in the process of female pregnancy play an important role, not only related to metabolic abnormalities and diseases but also causing the emergence of adverse outcomes [4]. Hypertensive disorders complicating pregnancy (HDCP) and gestational diabetes mellitus (GDM) are the two most common diseases of pregnant women during pregnancy. HDCP and GDM not only have an important impact on the health of the mother but also increase health risks to the normal development of the fetus in utero [5, 6]. It can even affect the barrier function of the placenta, thus altering the uptake and utilization of trace elements in the maternal-fetal system.

At present, due to the differences in various aspects, the changes in these trace elements in late pregnancy of Chinese pregnant women are not clear. Micronutrient deficiency in pregnant women and how it affects pregnancy outcomes are also unclear. However, studies have reported steady-state concentrations of trace elements in healthy maternal and umbilical cord blood [4, 7]. There are also few reports about trace elements in maternal and fetal systems of HDCP and GDM. Therefore, the present study intended to evaluate the levels and distribution of Zn, Cu, Fe, Mn, Ni and Cr in the maternal and fetal systems of patients with HDCP and GDM and to compare the HDCP and GDM groups with the normal group (full-term pregnancy and pregnant women without complications), and to analyze the characteristics of maternal to fetal metastasis of different trace elements in healthy individuals and patients with HDCP and GDM.

## Materials and methods

### Research object and grouping

In this study, a case–control method was used. The participants were all women who delivered in the Department of Obstetrics of The First People’s Hospital of Yunnan Province between January 2019 and December 2020. The inclusion criteria were as follows: (1) single pregnancy; (2) long-term residence in the study area; and (3) no familial hereditary diseases. Exclusion criteria included (1) having a history of special or occupational contact (e.g., Mining workers et al.); (2) having bad habits, such as smoking, drinking, or taking drugs; (3) illiterate (unable to sign informed consent); and (4) death or malformation of the newborn, stillbirth, twin or multiple births. In addition, an oral questionnaire and case survey were used to collect the basic demographic information of participants and information relating to newborn birth weight, birth length, and head circumference. After strict screening, a total of 140 pregnant women (normal group: 60 cases, HDCP group: 40 cases, GDM group: 40 cases) meeting the research conditions were included. The study protocol was approved by the Ethics and Research Committee of Yunnan First People’s Hospital and was conducted by the Declaration of Helsinki. Each participant was informed of the complete description of the study and informed consent was signed before enrollment.

The participants in this study included 140 pregnant women and their newborns, including 60 pregnant women who were healthy in all physical indicators of pregnant women, had no gestational diseases, and delivered at full term. Pregnant women with HDCP were divided into the group of hypertensive disorders complicating pregnancy (HDCP group): 40 cases. Pregnant women with GDM were divided into a gestational diabetes group (GDM group): 40 cases.

### Paired sample collection and processing

Paired sample collection was performed to collect 1 tube of maternal venous blood (3 ml/tube), 1 tube of umbilical cord blood (3 ml/tube) and a piece of placental tissue (cut from the edge of the placenta to the midpoint of umbilical cord attachment on the fetal side, 5 g/piece) immediately after delivery. The collected samples were labeled with corresponding information and loaded into the same bag together with placental tissue samples. The samples were transported to the laboratory refrigerator and frozen at -80 °C for subsequent use. During laboratory analysis, the sample was thawed at 4 °C, washed with deionized water three times, and then dried with a clean paper towel to remove excess water. One gram of wet placental tissue, 5 ml of nitric acid (BV grade III), and 2 ml of H_2_O_2_ were mixed and digested by a high-pressure microwave. A 0.5 g blood sample, 3 ml nitric acid (BV grade III) and 1 ml H_2_O_2_ were mixed and digested by a high-pressure microwave.

The working procedure of microwave digestion was set as follows: the power of the digestion system in the first stage was 16 W, the temperature was 120 °C, the temperature was raised for 5 min, and the temperature was maintained for 5 min. In the second stage, the temperature was increased to 140 °C for 5 min and maintained for 5 min. In the third stage, the temperature was increased to 160 °C for 5 min and maintained for 10 min. In the fourth stage, the temperature was increased to 180 °C for 5 min and maintained for 20 min. Finally, when the temperature dropped to room temperature, the digested solution was diluted to 25 ml with 1% HNO_3_.

### Detection and analysis of trace elements

The treated samples were analyzed by inductively coupled plasma–mass spectrometry (ICP–MS, Nexion 350x, USA). Certified standard 100 from Aladdin µg/ml was used as the multielement standard solution, and the standard curve was established. An inductively coupled plasma mass spectrometer (ICP–MS) was calibrated with indium, scandium, and rhenium as internal parameters. Two blank solutions were prepared for each analysis.

### Statistical analysis

The statistical software used was SPSS (Windows, version 17.0). Independent sample T-tests were used to evaluate whether there were significant differences in trace element levels in different groups (Normal group, HDCP group and GDM group). The correlations were analyzed based on Pearson’s correlation factor (r). *P* < 0.05 was considered statistically significant.

## Results

### Maternal characteristics and neonatal parameters

After statistical analysis, the maternal characteristics and neonatal parameters of the disease group (n = 80) and the normal group (n = 60) are listed in Table 1. There was a significant difference in maternal age between the normal group and the disease group (*P* < 0.001).

**Table 1:** Comparison of maternal and neonatal characteristics between disease group and normal group

	General characteristics	Normal group	Disease groups					
			HDCP group	Normal *&* HDCP *P* -Value	GDM group	Normal *&* GDM *P* -Value	Total	Normal *&* Disease *P* -Value
	Sample numbers	60	40		40		80	
Maternal	Age (years)	29.40 ± 3.36	30.95 ± 4.55	0.053	32.55 ± 3.92	< 0.001	31.75 ± 4.30	< 0.001
	BMI (kg/m^2^)	26.23 ± 2.61	27.55 ± 3.99	0.080	26.69 ± 2.27	0.374	27.11 ± 3.23	0.103
	Weight (kg)	68.11 ± 7.79	70.30 ± 11.68	0.318	67.56 ± 6.37	0.721	68.90 ± 9.38	0.615
Neonatal	Gestational age(weeks)	39.58 ± 1.02	37.60 ± 2.18	< 0.001	38.70 ± 2.78	0.126	38.34 ± 2.35	< 0.001
	Weight (kg)	3.32 ± 0.34	2.85 ± 0.65	< 0.001	3.31 ± 0.47	0.633	3.07 ± 0.60	0.002
	Height (cm)	50.69 ± 1.4	47.70 ± 4.01	< 0.001	50.21 ± 2.17	0.119	48.93 ± 3.40	< 0.001
	Apgar score in 1 min	9.23 ± 0.53	8.90 ± 0.30	< 0.001	8.97 ± 0.16	< 0.001	8.94 ± 0.24	< 0.001
	Apgar score in 5 min	9.98 ± 0.13	9.80 ± 0.46	0.019	9.97 ± 0.16	0.773	9.89 ± 0.36	0.028
	Adverse outcome rate (%)	3.33	10.00		10.00		10.00	

The values reported in the table are mean ± standard error. *P* < 0.001 and *P* < 0.05 indicated a significant difference, while *P* > 0.05 indicated no significant difference

The Apgar score provides an overall overview of neonatal health. As shown in Table 1, the Apgar scores of newborns in the disease group at 1 and 5 min were significantly lower than those in the normal group (*P* < 0.05). At the same time, the probability of adverse outcomes in the disease group was 10.00%, which was higher than 3.33% in the normal group.

In the study, we determined correlations between the 6 elements in the Mother blood, placenta, umbilical cord blood, and the neonatal Apgar scores 1 and 5 min. The results are presented in Table 2.

**Table 2:** Correlation between trace elements and neonatal Apgar score

Element	Sample	Normal group(n = 60)		HDCP group(n = 40)		GDM group(n = 40)		Total disease group(n = 80)	
		1 min	5 min	1 min	5 min	1 min	5 min	1 min	5 min
Zn	Mother blood								
Cu									
Fe									
Mn				-0.4362**				-0.2247*	
Ni									
Cr		-0.4003**							
Zn	Placenta				-0.4284**			-0.2785*	
Cu									
Fe		-0.3522**				0.3181*	0.3181*		
Mn		0.3009*							
Ni									
Cr		-0.4064**		-0.5931****	-0.6713****			-0.4294****	-0.5456****
Zn	Umbilical cord blood								
Cu									
Fe		-0.2919*							
Mn		0.2943*							
Ni									
Cr		-0.4024**							

The values reported in the table are Pearson r. Significant *P* values are 0.12 (ns), 0.0332 (*), * * 0.0021 (* *), 0.0002 (* * *), < 0.0001 (* * * *)

### Distribution characteristics of trace elements in the maternal-fetal system

**Fig. 1:**
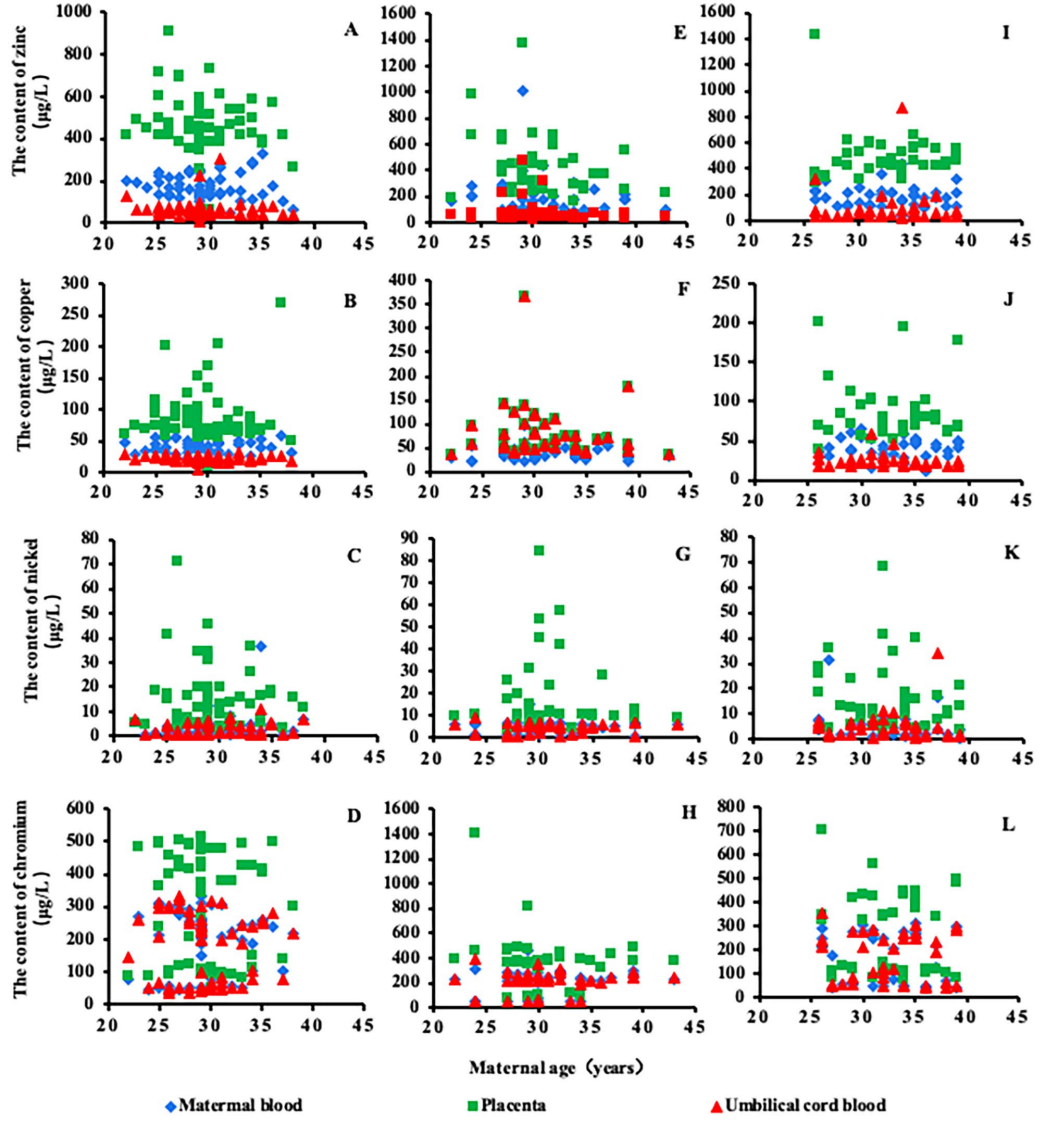
Distribution of trace elements Zn, Cu, Ni, and Cr in the maternal-fetal system. (Fig. 1**A-D**): Normal group (Fig. 1**E-H**): HDCP group (Fig. 1**I-L**): GDM group

The content distribution of trace elements Zn, Cu, Ni and Cr detected in the maternal-fetal system in the three groups is shown in Fig. 1. The average content distribution of Zn and Cu in the maternal-fetal system of the normal group was as follows: placenta > maternal blood > umbilical cord blood (*P* < 0.001). The distribution of the HDCP group and GDM group was the same as that of the normal group (*P* < 0.05). The average content distribution of Cr and Ni in the maternal-fetal system of the normal group was as follows: placenta > maternal blood > umbilical cord blood, and there were significant differences in Cr and Ni between the placenta, maternal blood and umbilical cord blood (*P* < 0.001). It is worth noting that the effectiveness of the placental barrier was shown by a higher Ni concentration in maternal blood than in umbilical cord blood (e.g., a ratio of maternal blood to umbilical cord blood > 1). The effective placental barrier ratio of Ni in the normal group was 60%, slightly higher than 55% in the HDCP group and 10% in the GDM group. In addition, the distribution and characteristics of the HDCP group and GDM group were the same as those of the normal group (*P* < 0.05).

**Fig. 2:**
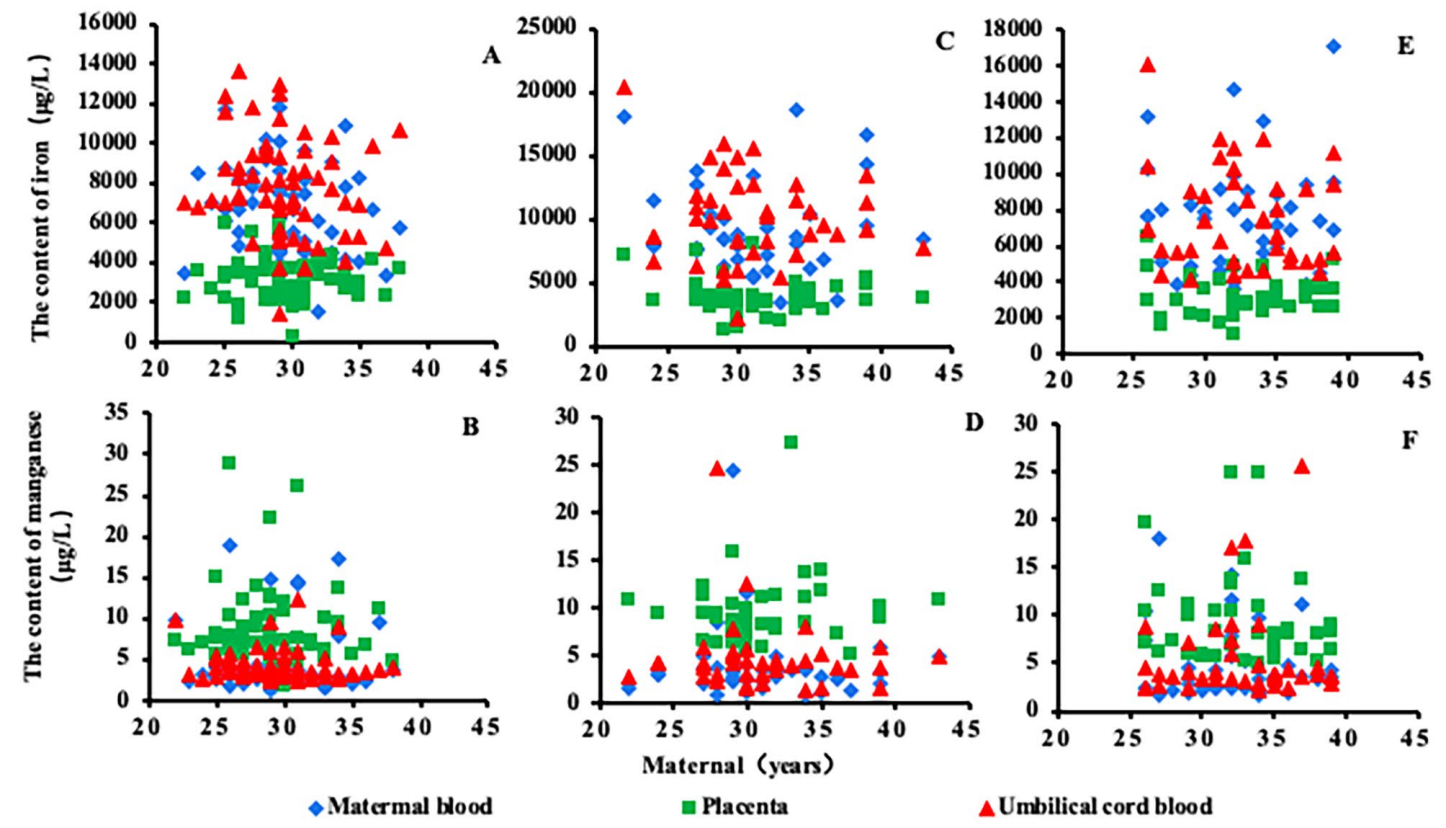
Distribution of trace elements Fe and Mn in the maternal-fetal system. (Fig. 2**A, B**): Normal group (Fig. 2**C, D**): HDCP group (Fig. 2**E, F**): GDM group

As shown in Fig. 2, the average distribution of Fe content in the maternal-fetal system of the normal group was as follows: umbilical cord blood > maternal blood > placenta (*P* < 0.05). However, the average distribution of Fe content in the HDCP group was cord blood > maternal blood > placenta. The average distribution of Fe content in the GDM group was maternal blood > umbilical cord blood > placenta. There was no significant difference in Fe content between maternal blood and umbilical cord blood between the HDCP group and GDM group (*P* > 0.05). The average distribution of Mn content in the maternal-fetal system of the normal group was as follows: placenta > maternal blood > umbilical cord blood. There was a significant difference in Mn content between the placenta, maternal blood and umbilical cord blood (*P* < 0.001). Distribution characteristics of the HDCP group and GDM group were as follows: placenta > umbilical cord blood > maternal blood. There was a significant difference in Mn content between the placenta, maternal blood and umbilical cord blood in the HDCP group (*P* < 0.05), while there was only a significant difference in Mn content between the placenta and maternal blood in the GDM group (*P* < 0.001).

### Differences between the disease group and the normal group

**Fig. 3:**
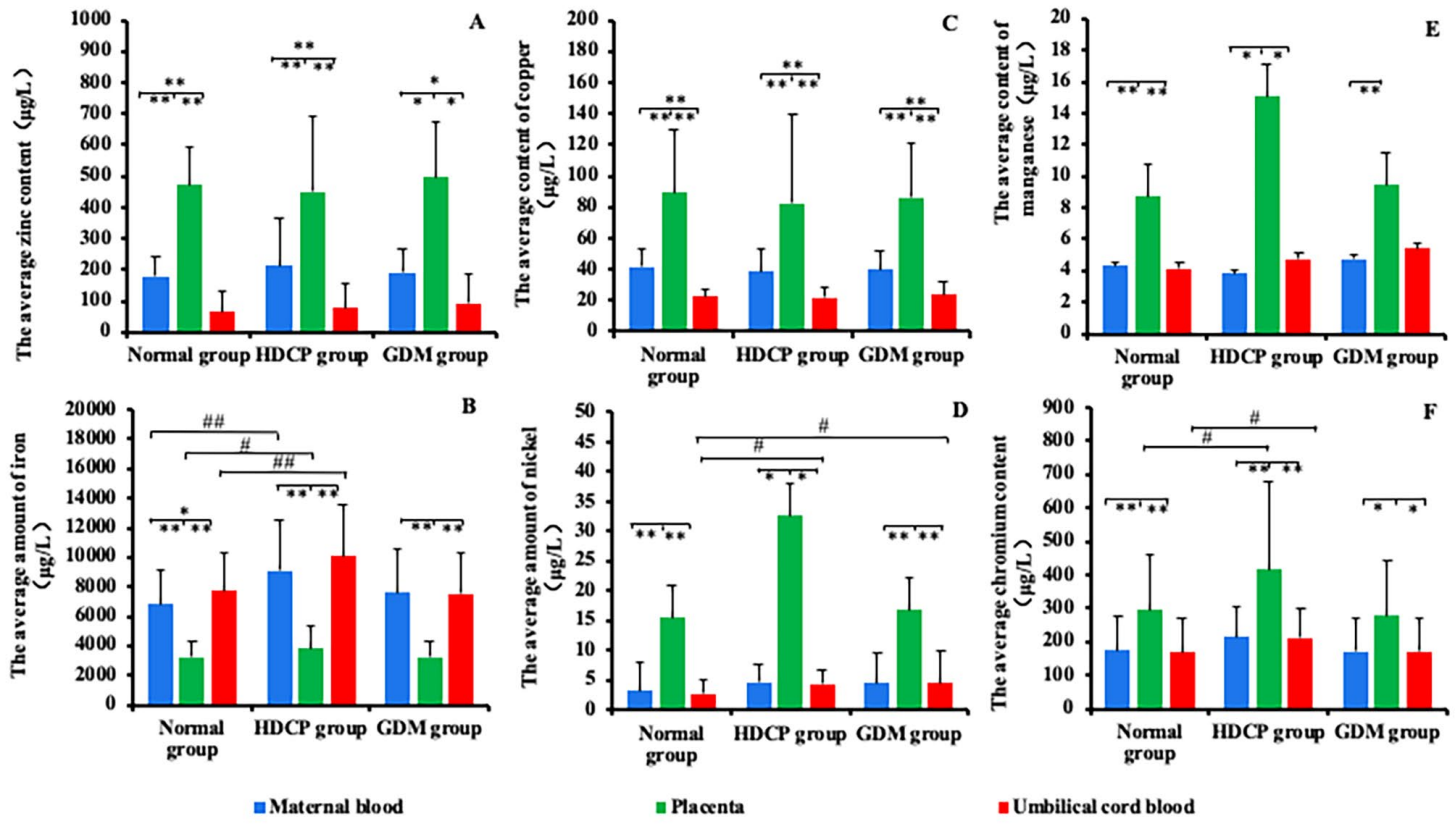
The contents of trace elements in maternal blood, placenta, and umbilical cord blood of the normal and disease groups. (Fig. 3**A**): Zn, (Fig. 3**B**): Fe, (Fig. 3**C**): Cu, (Fig. 3**D**): Ni, (Fig. 3**E**): Mn, (Fig. 3**F**): Cr. (* and ** indicate that there are significant differences among matched samples in the same group, *P* < 0.05 and *P* < 0.001; #, ## indicate that there are significant differences among the same samples in different groups, *P* < 0.05, *P* < 0.001.)

Figure 3 shows the contents of trace elements in the blood, placenta and umbilical cord blood of pregnant women in the normal group and the disease group. In the HDCP group, it was observed that Fe in maternal blood, placenta and umbilical cord blood was higher than that in the normal group (*P* < 0.05). In the HDCP group, Cr was higher in placenta and umbilical cord blood than in the normal group (*P* < 0.05). Although the average content of Cr in the maternal blood of the HDCP group was also higher than that of the normal group (Table 3), there was no significant difference (*P* > 0.05). Ni in cord blood of the HDCP group and GDM group was also higher than that in cord blood of the normal group (*P* < 0.05). As shown in Table 3, there was no significant difference in the contents of other trace elements (such as Zn, Cu and Mn) between the maternal-fetal system of the normal group and the disease group (*P* > 0.05).

**Table 3:** Trace element level of mother blood, placenta and umbilical cord blood

Element	Sample	Normal group		HDCP group		Normal *&* HDCP *P* - Value	GDM group		Normal *&* GDM *P*- Value
		Mean ± SD	Min - Max	Mean ± SD	Min - Max		Mean ± SD	Min - Max	
Zn	Mother blood (µg/L)	180.35 ± 61.67	46.11-333.48	212.35 ± 156.27	60.70-1006.21	0.224	191.31 ± 77.56	33.33-444.28	0.435
Cu		41.73 ± 11.59	14.69–72.87	38.27 ± 14.71	21.24–97.37	0.193	39.66 ± 12.46	11.24–66.34	0.398
Fe		6834.92 ± 2298.02	1557.99-12248.97	9095.75 ± 3510.55	3487.98-18590.35	< 0.001	7587.09 ± 3064.02	2776.12-17040.96	0.164
Mn		4.29 ± 3.89	1.12–19.06	3.82 ± 3.89	0.83–24.57	0.553	4.72 ± 3.73	1.55–17.91	0.582
Ni		3.22 ± 4.92	0.50-36.48	4.69 ± 2.88	0.54–15.30	0.093	4.48 ± 5.35	0.55–31.04	0.231
Cr		175.59 ± 102.30	44.55-330.32	214.25 ± 91.79	47.25-457.87	0.051	171.20 ± 103.22	39.35-308.97	0.834
Zn	Placenta (µg/L)	471.01 ± 125.13	65.70-908.79	449.37 ± 246.51	164.75-1373.54	0.610	495.97 ± 179.88	301.76-1440.29	0.415
Cu		89.38 ± 40.61	8.82-270.21	82.44 ± 57.29	36.37-367.86	0.480	85.87 ± 35.74	39.99-200.38	0.658
Fe		3252.93 ± 1073.52	320.24-6026.68	3829.27 ± 1495.36	1265.59-8080.42	0.027	3231.34 ± 1089.47	1094.12-6558.58	0.922
Mn		8.71 ± 4.77	1.83–28.92	15.05 ± 23.43	5.10-114.64	0.099	9.46 ± 4.82	5.10-24.79	0.442
Ni		15.57 ± 12.48	1.61–70.84	32.68 ± 64.13	3.43-299.15	0.103	16.77 ± 13.34	3.41–68.44	0.647
Cr		295.93 ± 165.75	53.63-515.06	414.66 ± 268.01	83.75-1404.87	0.007	277.71 ± 169.43	77.89–703.60	0.595
Zn	Umbilical cord blood (µg/L)	64.77 ± 43.21	11.07-308.85	78.21 ± 87.24	22.83-471.54	0.371	92.30 ± 139.20	26.72-873.66	0.232
Cu		22.41 ± 4.47	4.28–32.01	21.89 ± 6.58	11.66–43.22	0.663	23.96 ± 7.87	16.71–57.84	0.211
Fe		7758.87 ± 2508.22	1425.04-13555.11	10071.61 ± 3509.84	2165.79-20408.09	< 0.001	7562.75 ± 2731.44	4068.62-16111.09	0.712
Mn		4.15 ± 1.93	2.34–12.22	4.72 ± 3.84	1.33–24.78	0.331	7.36 ± 13.53	2.07–25.62	0.144
Ni		2.71 ± 2.27	0.34–11.24	4.43 ± 2.31	0.64–9.30	< 0.001	4.52 ± 5.53	0.55–34.05	0.025
Cr		168.60 ± 103.99	35.35-331.21	210.65 ± 88.81	46.71-394.55	0.033	170.47 ± 101.30	40.57-357.44	0.929

The values reported in the table are mean ± standard error. *P* < 0.001 and *P* < 0.05 indicated a significant difference, while *P* > 0.05 indicated no significant difference

## Discussion

### Iron barrier and transport in the placenta during pregnancy complications

As an effective component of hemoglobin, Fe is rich in maternal blood and umbilical cord blood. The placenta is the key hub for the transfer of Fe from the mother to the fetus. The movement of Fe in the maternal-fetal system is unidirectional - from the mother to the fetus. Therefore, the level of Fe on the fetal side does not change the dynamic characteristics of Fe absorption into the placenta [8]. Studies have shown that GDM-mediated hyperglycemia can affect placental Fe homeostasis [9]. Our study observed a negative correlation between placental Fe and Apgar score in the normal group, but this was reversed in the GDM group, and placental Fe had a higher promoting effect on Apgar score (Table 2). In addition, the content and distribution of Fe in the maternal blood, placenta and umbilical blood in the GDM group (Fig. 2E) also changed compared with those in the normal group(Fig. 2A). Fe can be used as an oxidant to participate in the pathogenesis of GDM, its main function is to induce insulin resistance and interfere with insulin release from pancreatic beta cells [10]. Therefore, the abnormal distribution of Fe content in the maternal-fetal system in the GDM group may be mainly caused by the maternal side. More and more studies suggest that maternal Fe content is a risk factor for GDM [11]. The occurrence of GDM can also affect the barrier and transport function of the placenta about Fe, thus affecting the transfer efficiency of Fe in the maternal-fetal system and increasing the risk of fetal health.

Fe is the producer of free radicals (FRs) (oxidants), and Fe supplementation may put people at risk of excessive intake of Fe [12]. Indeed, the chemical properties of Fe give it potential harm. This is mainly due to the key role of Fe in the Fenton reaction, which leads to the production of highly toxic hydroxyl radicals (·OH) [13]. Since free-form sub-Fe ions (Fe^2+^) can cause the production of FR or reactive oxygen species (ROS), which can lead to peroxidation and FR chain reactions and finally to molecular damage [12]. A high Fe content will increase the risk of HDCP (e.g., PE) [14, 15]. It is worth noting that the content of Fe in maternal blood, placenta and umbilical cord blood in the HDCP group was higher than that in the normal group (Fig. 3B), and the results were consistent with previous literature reports [14, 16]. With the improvement of living standards and the enhancement of people’s awareness of health care, it is generally believed that the demand for Fe during pregnancy is very high, so Fe is traditionally used as a supplement during pregnancy. Although this may be correct in a few cases, most pregnant women do not need to take excessive Fe supplements [12].

### The occurrence of GDM affects the placental transport of Mn

Mn is an important trace element in the human body. Its normal level in the body is very important for the health of the mother and fetus [17]. This study observed that the content of Mn in the placenta in different groups was significantly higher than that in maternal blood, which mainly reflected the active transport of Mn from the mother to the placenta in the maternal-fetal system. In addition, studies have shown that Mn is involved in glucose metabolism in the human body and inhibits glucose interference with insulin secretion through the activation of related pathways, thus increasing the risk of GDM [18–20]. The content and distribution of Mn in maternal blood, placenta, and umbilical blood in the GDM group (Fig. 2F) were changed compared with that in the normal group(Fig. 2B). The occurrence of GDM may affect the placental transport of Mn and lead to the risk of excessive intake of fetal Mn. Studies have shown that high levels of Mn exposure can affect indicators of fetal development [21]. In addition, there was no significant difference in Mn content between the normal group and the GDM group (Fig. 3E), which was consistent with other research results [22–24].

### Pregnancy complications weakened the placental barrier function of Ni

The placenta plays an important role in the transfer of Ni from the mother to the fetus, although the placenta provides a protective barrier against Ni toxicity [25], However, with increased pregnancy duration, the ability of the placenta to absorb and retain Ni also increases [26]. Studies have shown that in the maternal-fetal system with gestational diseases, the transportation of Ni in the placenta will significantly change the morphology and permeability characteristics of the placenta during development [26]. The barrier and normal function of the placenta are damaged, as shown in Fig. 3D, which shows that the Ni content in the cord blood of the HDCP group and GDM group was significantly higher than that of the normal group (*P* < 0.05). Therefore, we speculate that Ni in the HDCP group and GDM group can not only accumulate in the placenta but also damage the placental barrier function of Ni so that more Ni can pass through the placenta and enter the fetal side. In addition, Ni hurts glucose metabolism, and the increase of maternal Ni content is associated with the risk of GDM [27], but the relevant evidence is limited, and more studies are needed to verify and explore its pathogenesis.

### Abnormal cr transport in HDCP and GDM maternal-fetal systems

Carbohydrate residues were found in the placental structure of normal and healthy pregnant women [28]. The abnormal morphology and function of the placenta in pregnant women with HDCP will lead to changes in carbohydrate morphology [29]. It has been documented that Cr is involved in the metabolism of carbohydrates and lipids [30]. HDCP may affect placental function by altering the placental structure, resulting in an imbalance of Cr transport in the maternal-fetal system, resulting in an increased risk of fetal Cr exposure (Fig. 3F). Abnormal Cr content can increase the rate of spontaneous abortion, affect fetal intrauterine growth and increase the risk of adverse outcomes [31]. The correlation analysis between trace elements and the Apgar score showed that Cr content in the placenta was negatively correlated with the Apgar score(Table 2). Apgar scores are associated with an increased risk of neurological and cognitive impairment in early adulthood [32]. In addition, Cr also plays a very important role in the process of glucose metabolism, but no significant correlation was found between Cr content and the risk of GDM in this study, which is consistent with the results of other case-control studies [33, 34].

### Zinc and copper in maternal-fetal systems

Zn and Cu are important trace elements in the human body and are very important for maternal health and fetal development. Zn and Cu can be used as micronutrients, which play a positive role in maternal and fetal health, and as antioxidant elements, which participate in oxidative stress processes [35, 36]. Oxidative stress is one of the key pathogenesis of pregnancy diseases (e.g., PE). The results of this study show that the content and distribution of Zn and Cu in the pregnancy disease group (Fig. 1E-F, I-J) were not significantly different from those in the normal group (Fig. 1A-B). It can be seen that Zn and Cu have little or no change in homeostasis in the maternal-fetal system of HDCP and GDM. It has also been reported that there are a large number of enzymes containing Zn and Cu in the placenta itself [37–39]. The neutralization of physiological and pathological mechanisms may explain this result.

### Strengths and limitations of the study

To the best of our knowledge, this is one of the few studies in which collected paired samples of maternal blood, placenta, and umbilical cord blood from both normal and disease groups. We observed that the distribution characteristics of trace elements (Zn, Cu, Fe, Mn, Ni, Cr) in the maternal-fetal system and related to HDCP and GDM. Our work also has its limitations: an insufficient number of participants was in each group. The previous evidence is limited and contradictory, and the etiology of HDCP and GDM and the factors affecting the transfer of trace elements are diverse. Therefore, we only performed a preliminary analysis of the distribution characteristics of trace elements in the maternal and fetal systems of HDCP and GDM and did not further examine how trace elements increase health risks. The interaction of HDCP and GDM with trace elements in the maternal-fetal system needs further investigation.

## Conclusion

This study is one of the few studies to detect and evaluate the levels of trace elements in the maternal-fetal system (maternal blood, placenta, and umbilical cord blood) of HDCP and GDM. This study found that the transport and distribution characteristics of Fe, Mn, Ni and Cr in the maternal-fetal system of HDCP were changed. The transport and distribution characteristics of Fe, Mn and Ni in the maternal-fetal system of GDM were changed. Among them, the levels of HDCP placenta Cr and GDM placenta Fe were correlated with the Apgar score. Identifying the unique and subtle changes in trace elements can provide a deep understanding of the pathophysiological characteristics of pregnancy complications to explore the pathogenesis and risk factors of HDCP and GDM. At the same time, this study also provides a valuable basis for the clinical prevention of HDCP and GDM, and the improvement of maternal and fetal health.

## Data Availability

All data requests should be submitted to the corresponding author for consideration. Access to anonymized data may be granted following review.

## Abbreviations

Zn: Zinc
Cu: Copper
Fe: Iron
Mn: Manganese
Ni: Nickel
Cr: Chromium
HDCP: Hypertensive disorder complicating pregnancy
GDM: Gestational diabetes mellitus
ICP-MS: Inductively coupled plasma - mass spectrometry
FRs: Free radicals
ROS: Reactive oxygen species
